# Digital educational games in veterinary education: a scoping review with comparison to human medical education

**DOI:** 10.3389/fvets.2026.1929108

**Published:** 2026-10-14

**Authors:** Ingrid Toftaker, Karin Berggren Gustafsson, Lena-Mari Tamminen

**Affiliations:** 1 Department of Production Animal Clinical Sciences, Norwegian University of Life Sciences, Ås, Norway; 2 Department of Clinical Sciences, Swedish University of Agricultural Sciences, Uppsala, Sweden

**Keywords:** education, gamification, learning, problem-based learning, serious games, students

## Abstract

**Inroduction:**

Game-based learning, including approaches such as serious games and gamification, is used to enhance engagement and learning in education. While its use in medical education has been reviewed, its application in veterinary education remains underexplored. This scoping review summarizes the use of digital game-based learning in veterinary medicine.

**Methodology:**

Searches of Web of Science, Medline, and Scopus were conducted, following the Population/Concept/Context framework and PRISMA-ScR guidelines. Thirteen veterinary education publications were identified and compared to 177 human medical education articles.

**Results:**

In both fields, games were mainly used in clinical sciences, with common types including simulations and case-based learning covering diverse topics. Ten veterinary studies included evaluations, with six assessing learning outcomes, and eight examining affective outcomes such as motivation and confidence. Cognitive findings were mixed, while some studies reported positive effects on learning outcomes, others found no significant differences, and one study reported lower scores in part of the assessment for the intervention group. Control groups that did not interact with the game were included in only two of the veterinary studies and only one used a systematic allocation procedure. Access to games was often poorly documented.

**Discussion:**

Overall, the included studies suggest that game-based learning is generally well-received and may enhance learning, although one study reported low uptake of the game activity. However, further research is needed to determine the conditions that optimize its effectiveness.

**Systematic review registration:**

https://osf.io/cnpk5/overview.

## 1 Introduction

The approaches to teaching in higher education are constantly evolving, and in several fields there is a shift from didactic teaching methods towards a more student-active approach, where the term student-active learning is used to encompass all teaching formats in which students are actively engaged in their own learning process (1). In several medical related fields, the implementation of student-centered active learning pedagogy is now a required component (2, 3). This is also the case in veterinary medicine as emphasized by the accreditation policies, including the European System of Evaluation of Veterinary training (4). In Europe, evaluation and approval of veterinary training is managed by the European Association of Establishments for Veterinary Education (EAEVE) in association with the Federation of Veterinarians of Europe (FVE).

A review of veterinary pedagogy stated that student-active learning is crucial to effective educational progress, listing it as one of nine pillars for building a modern veterinary curriculum (1). From our experience, many veterinary students, particularly in the early stages of their education, envision themselves as future veterinary practitioners. However, before they can engage fully in clinical training, they must first undergo rigorous foundational training in subjects such as biochemistry, physiology, and epidemiology. These subjects can often feel overly theoretical, making it difficult for students to see their practical applications.

Incorporating student-active learning approaches is one strategy to help students recognize the relevance and applicability of these foundational concepts. On the other hand, student-active learning methods might be perceived as more demanding by students compared to passive listening. Nevertheless, a systematic review of the students affective responses to active learning, reported that students were positive in the vast majority of included studies (5). In health-care related disciplines, Harris et al. (6) reported that student-active methods are beneficial for improving students’ knowledge, understanding, and application of information gained in didactic settings. Similarly, in veterinary education, student-active learning methods have been shown to enhance affective outcomes. For instance, in a study from Berrian et al. (7) the students reported increased competency and self-efficacy of practical skills. Additionally, student-active methods have been identified as effective in veterinary epidemiology, where they foster student engagement and support deeper learning (8).

Among the student-active learning methods that have gained popularity in recent years is the use of *game-based learning* (GBL) activities. Put simply, game-based learning can be defined as *learning that is facilitated by the use of a game* (9). By incorporating elements commonly associated with gameplay, such as competition, rewards, and problem-solving, GBL aims to enhance student engagement in the learning process. A wide variety of different GBL activities have emerged, such as various simulation activities, quizzes and card games. The related term *serious gaming* is often used to describe digital games specifically designed for educational or training purposes, rather than for pure entertainment (10, 11), while the term gamification is used to describe the incorporation of game elements in a nongame context (12, 13).

To maximize the benefit of any learning activity, including GBL activities, it is essential that the activity aligns with the intended learning outcomes of a course, following the principle of *constructive alignment* (14). An important advantage of GBL in veterinary education is the opportunity it provides for students to practice critical skills, such as clinical decision-making and problem-solving, by simulating real-world scenarios. This can be used as a supplement to the resource demanding field training and training that involves interacting with live animals. In this way, implementation of GBL does not only support alignment between learning activities and learning outcomes of individual courses but also helps prepare students for challenges they may face later in their professional careers. Several reviews and meta-analysis have explored the implementation of game-like activities in educational settings (15–17). Two recent reviews specifically examined the use of GBL within the education of human health professionals (18, 19), highlighting the potential of GBL to enhance learning outcomes, with these activities generally being well-received by learners. However, Xu et al. (19) emphasize that many studies do not seek to understand the underlying mechanisms through which specific game elements contribute to learning. Despite the growing interest in GBL, research on its application within veterinary education remains limited, leaving a gap in our understanding of its potential benefits in this field.

The present study undertakes a scoping review of research describing the use of digital GBL in the veterinary medical field. A secondary, contextual objective was to compare the use in veterinary education to the human medical education. We formulated the following research questions: For studies of digital educational games in veterinary medicine; what type of games are reported, how are GBL activities evaluated, including what outcomes are measured, and is information about software and game accessibility provided? As a contextual background: How does the number of reports in veterinary medical education compare to the use in human medical education? How much use is reported at the undergraduate compared to the postgraduate level in the two disciplines?

## 2 Methods

### 2.1 Protocol and registration

The Preferred Reporting Items for Systematic Reviews and Meta-Analysis for Scoping Reviews (PRISMA-ScR) were used in the planning and reporting of this review (20). We also used the recommendations from Pollock et al. (21) to guide the review process. To gain an initial understanding of the existing literature, preliminary searches were conducted. Based on these findings, a protocol outlining the objectives, eligibility criteria, and methods was developed and registered on the Open Science Framework (https://doi.org/10.17605/OSF.IO/CNPK5).

### 2.2 Eligibility criteria

We aimed to search for papers describing *the use of game-based learning activities using a digital device in educational contexts*. As emphasized by van Gaalen (18), there is a lack of consensus in the literature both regarding what a game is and the essential elements of a game, thus uniformly accepted definitions are hard to find. We defined *a game* building on the definition from Salen and Zimmerman (22) who describes a game as “a game is a system in which players engage in an artificial conflict, defined by rules, that results in a quantifiable outcome”. However, as mentioned by Adams (23), requiring an “artificial conflict” might be too restrictive and cause, e.g., cooperative games to be excluded. We therefore chose the simplified definition *an activity with some rules engaged in for an outcome*. Furthermore, different authors make suggestions of various elements that are often included in games (22–24). Based on this, the elements that we chose to look for were (1) *rules*, which define how the game is played and establish structure, (2) *goals*, many games have a definition of a specific goals that players aim to reach, (3) *interaction with others*, either as competition or team effort, or against the system, (4) *challenge*, typically involving some sort of problem solving and (5) *feedback or outcome*, i.e., some sort of point scoring, achievement or feedback to players. At least two of these elements had to be present for the activity to be classified as a game. We deliberately adopted a broad inclusion approach, consistent with the aims of a scoping review, in order to capture the range of relevant studies in a field where terminology is inconsistent and overlapping. Furthermore, we only included digital games meaning the game had to use some sort of digital platform, such as PC, tablet or phone. A game was also eligible if it combined a digital platform with the physical space (virtual and augmented reality games), but physical games with no digital platform were excluded (e.g., role play and board games). As stated by Salen and Zimmerman (22), digital games can take a multitude of forms and appear on many different computer platforms, and we chose to include articles describing games that used any kind of digital device, not limiting the definition to specific systems (software or hardware). The definitions used in the current study are listed in Table 1.

**Table 1:** Definitions of four key terms used to outline the eligibility criteria and for labelling of publications in scoping review of digital game-based learning in veterinary education.

Term	Definition
Game	An activity with some rules engaged in for an outcome containing elements of game playing (rules, goals, interaction with others, challenge and feedback or outcome).
Digital game	A game that is played using a digital device (e.g., computer, TV, phone, tablet), including virtual- and augmented reality.
Learning	The process of learning something, i.e., acquiring new knowledge or skills.
Simulation	A situation in which a particular set of conditions is created artificially in order to study or experience something that could exist in reality (40).

In this review, we included only peer-reviewed scientific articles that were full research papers, published with both an abstract and full text available in English. To be eligible, articles had to describe original research, excluding conference proceedings, review articles, editorials, and opinion pieces. There were no restrictions on the publication date. Articles were required to be either open access or accessible through the university library subscription services of the Swedish University of Agricultural Sciences or the Norwegian University of Life Sciences. Studies were excluded if they were not peer reviewed, were not full primary research articles, were not published in English, did not include the target population, did not meet our definition of a digital game, or did not use the activity for learning purposes.

Included articles needed to describe the use of a digital game in an educational context. This included games utilized in undergraduate or graduate education, as well as in continuing education for licensed physicians or veterinarians, hereafter referred to as *postgraduate education*. In line with the aims of this review, studies from human medical education were included for comparison. However, given the focus of this review on veterinary medicine, articles describing digital educational games in veterinary education are described in greater detail (see sections “Data Charting Process” and “Data Items”).

An overview of the inclusion and exclusion criteria for this review is provided in Table 2.

**Table 2:** Inclusion and exclusion criteria for selection of studies.

Criterion	Inclusion	Exclusion
Databases	Web of Science, Medline, Scopus	Other databases (not searched)
Journal	Peer reviewed journal	Not peer reviewed
Publication types	Primary research, full paper	Review articles, conference proceedings, editorials
Language	English	Other languages
Population	Medical or veterinary students or practitioners	Others
Digital game	Description of activity fulfilling the definition of a digital game (Table 1)	Description of activity not fulfilling the definition of a digital game (Table 1)
Purpose	Used for learning	Used for other purposes

### 2.3 Information sources

For this scoping review, we performed a literature search on 20 February 2024 in the three databases: Web of Science, Scopus and Medline. Additionally, we checked the reference lists for all included papers to identify any further relevant articles.

### 2.4 Literature search

According to our research questions, we developed the search strategy based on the PCC (Population (or participants)/Concept/Context) framework, which can be used to construct clear and meaningful objectives and eligibility criteria for a scoping review (21). An overview of the search strategy and search terms can be found in Table 3.

**Table 3:** Search terms and combinations using the PCC (Population (or participants)/Concept/Context) framework.

PCC element	Search block	Search terms
Population/Participants	1	teacher* OR student* OR practitioner*
Concept	2	game* OR gami*
	3	pedagog* OR didactic* OR teach* OR learn* OR educa*
Context	4	Serious OR simulat* OR virtual OR immers* OR digital*
	5	medicine OR veterinar* OR medical

Search blocks were combined using AND to construct the final search.

Search results were exported into Rayyan (25), a reference tool that facilitates collaboration among the reviewers. The complete search for one database (Web of Science) is provided here:

Web of Science Core Collection (date: 20.02.2024) - 664 articles: TS = (game* OR gami*) AND TS = (teacher* OR student* OR practitioner*) AND TS = (pedagog* OR didactic* OR teaching OR learning OR educa*) AND TS = (Serious OR simulat* OR virtual OR immers* OR digital*) AND TS = (medicine OR veterinary OR medical).

### 2.5 Selection of sources of evidence

To ensure equal perception of the inclusion and exclusion criteria (Table 2) an initial calibration among all three authors was performed; all three authors individually screened 11 randomly selected papers (reading title and abstract) to assess inclusion blinded to the other authors assessment. The agreement among the authors was assessed in a joint meeting. The same inclusion decision was reached for nine of the 11 articles. The remaining two articles prompted discussion regarding the inclusion criteria, and these points were subsequently clarified. Following this, all papers were screened by a single author (KBG) who read the title and abstract to assess whether the paper met the inclusion criteria. In all cases of uncertainty, the papers were flagged and screened by all authors in joint meetings. Papers where the eligibility criteria could not be assessed from screening of title and abstract were forwarded to the next step, i.e., reading of full text. In cases where an abstract was not available, the full text was read, and if the full text was not obtainable, the article was excluded.

During the screening (title and abstract), the articles were given labels to address who the target population was (physicians, veterinarians), in which part of the curriculum the game was used (undergraduate, postgraduate) and the subject area the game was intended for (basic science, clinical science or public health). The classification of subjects was based on the Standard Operating Procedure (SOP) of the European System of Evaluation of Veterinary Training (4) with some modifications; “Clinical Sciences in companion animals” and “Clinical Sciences in food-producing animals” were grouped together and termed clinical sciences, and the category “Professional Knowledge” (soft skills, e.g., communication, team working skills, management skills) was deemed not fit for purpose and excluded as practicing these skills can be considered inherent to game play. Thus, we used the three categories: Basic sciences, clinical sciences and veterinary public health. For the articles that could not be classified from title and abstract, full text was retrieved.

### 2.6 Data charting process

Data charting was performed as a two-step process as shown in Figure 1. Firstly, country and year of publication were extracted for all papers, including papers from both veterinary and human medical education. Additionally, data charting was performed based on the three key items: target population, place in curriculum and subject area. Data charting of these items was performed also for papers from human medicine (i.e., population was physicians) if they otherwise fulfilled the inclusion criteria, i.e., digital game, and a game used for a learning process. If these three items could not be extracted from title and abstract, the full text was read. This was performed to be able to compare the overall use of games between veterinary and human medical education.

**Figure 1:**
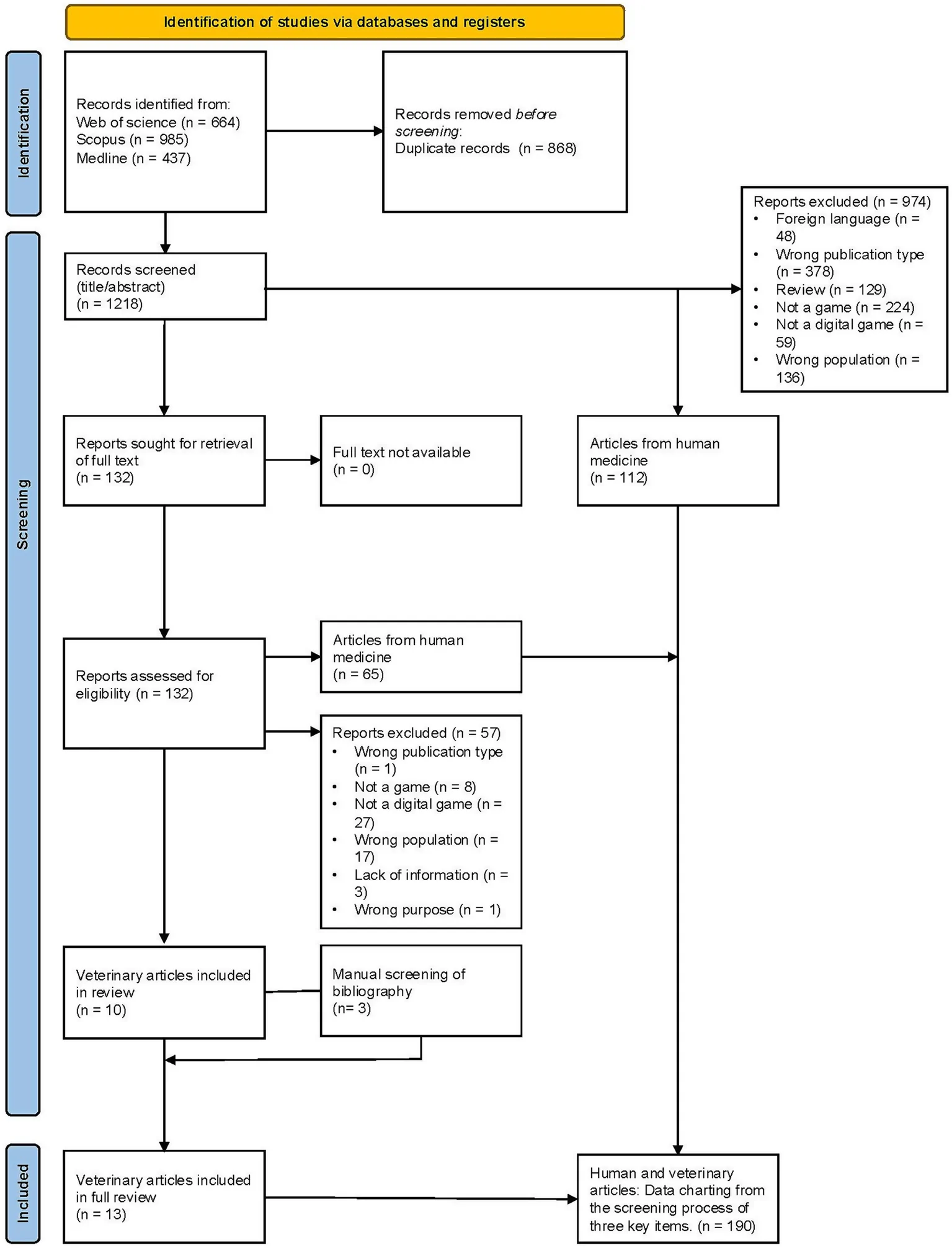
PRISMA flowchart describing the study selection process in a scoping review of digital educational games in veterinary medicine.

The second step was performed only for veterinary education articles and involved extracting additional data items. Full-text reviews of all veterinary articles were conducted, and data were systematically extracted using a Microsoft Form. The extracted data were then compiled and stored in Microsoft Excel (see Supplementary material). All authors reviewed the full texts of the included veterinary articles, and the data extraction process was discussed and refined during joint meetings to ensure consistency and accuracy.

### 2.7 Data items

For all publication in veterinary medicine, the following items were identified and extracted from full text:

1. General information about the article, e.g., author, title, publication year.
2. Name and short description of the game.
3. Details on the implementation in the curriculum: Undergraduate or postgraduate, which subject/course and which target skills to be learned.
4. Type of game, e.g., group work, case-based learning, simulators
5. Evaluation: Whether the article describes an evaluation of the learning activity. If so, details about the evaluation method (e.g., outcomes measured, sample size, and results).
6. Software: Software system used, if described.
7. Access and maintenance. Information on whether the game is maintained, and if it is accessible (e.g., via a provided link).

## 3 Results

### 3.1 Selection of sources of evidence

The search across three databases, Web of Science, Scopus, and Medline, yielded a total of 2,086 citations. After removing duplicates, 1,218 unique citations remained. Following title and abstract screening, full texts were retrieved for 132 citations. For 112 citations related to human medical education, all key data items could be extracted from the title and abstract alone, and full texts were therefore not retrieved.

After thorough assessment through abstract and full-text screening, 187 publications met the inclusion criteria for the initial data charting step. Of these, 10 articles were from veterinary medicine, while 177 were from human medicine. Additionally, three veterinary articles were identified through manual screening of the bibliographies of included veterinary papers. This brought the total number of veterinary articles included for detailed analysis to 13. A visualization of the process of identifying, screening, and selecting studies for inclusion in the review is presented in Figure 1.

### 3.2 General characteristics of sources of evidence

#### 3.2.1 Place in the curriculum and subject area

Of the 190 papers fulfilling the inclusion criteria, 13 were from veterinary medicine, and 177 were from human medicine. In both the human and veterinary medical education, most of the games were implemented in courses related to clinical science. An overview of the place in curriculum and subject area for all publications can be found in Table 4.

**Table 4:** Comparison of the number (%) of publications* describing digital educational games in higher education between veterinary medicine vs. human medicine, showing the subject area and place in curriculum for *n* = 190 articles identified in a scoping review of digital game-based learning in veterinary education.

Publication category	Veterinary medicine (*n* = 13)	Human medicine (*n* = 177)
Subject area		
Basic sciences	4 (30.8%)	45 (25.4%)
Clinical sciences	9 (69.2%)	127 (71.8%)
Public health	0 (0%)	6 (3.4%)
Place in curriculum		
Undergraduate level	12 (92.3%)	148 (83.6%)
Postgraduate level	1 (7.7%)	42 (23.7%)

^*^A publication could be classified into more than one category; therefore, the cumulative frequency exceeds 100%. In human medicine, one publication included several games and was classified as belonging to both Basic sciences and Clinical sciences, and 13 publications were classified as involving both undergraduate and postgraduate levels.

#### 3.2.2 Country and year of publication

We observed a large increase in the number of articles published on digital educational games in both human and veterinary medical education over the past decade. Notably, 171 out of 190 papers (90.0%) were published after January 1, 2014 (Figure 2A). The majority of first authors in both fields were affiliated with institutions in the United States (*n* = 39 papers, 20.5%). Additionally, the top five countries represented, all located in either Europe or North America, accounted for 96 papers (50.5% of the total). The geographical distribution of first author affiliations across all included articles is shown in Figure 2B.

**Figure 2:**
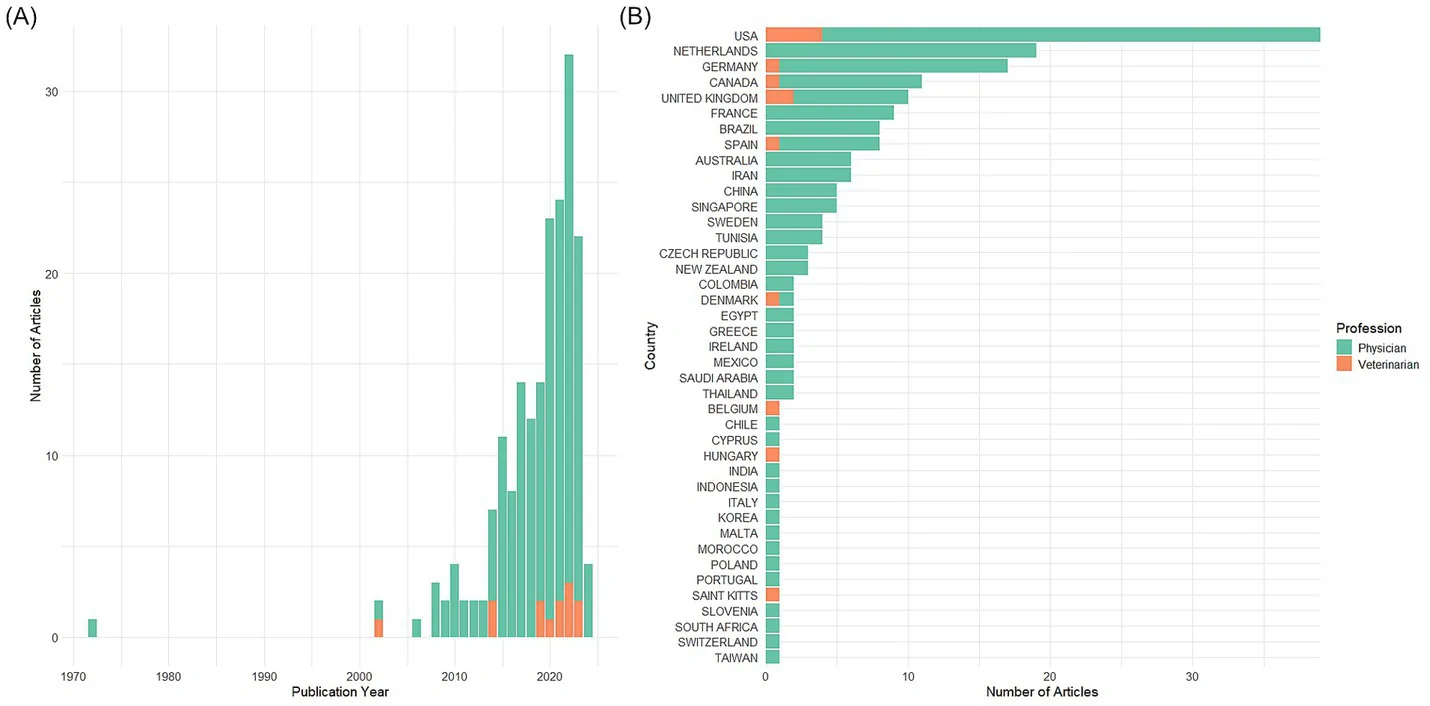
Distribution of frequency of publication year **(A)** and geography **(B)**, based on the affiliation of the first author, of articles on digital educational games for physicians (*n* = 177) and veterinarians (*n* = 13) included in a scoping review of digital educational games in veterinary medicine.

### 3.3 Results of individual sources of evidence

The extracted data items from the 13 publications in veterinary medicine, including the predefined game elements used to classify each activity as a game, are summarized in Table 5. The most commonly reported game types were simulations and case-based learning, with some games fitting into both categories. Clinical sciences were the most common subject area (9/13); however, the reviewed games spanned a diverse range of topics, including basic sciences such as neuropathology and physiology, as well as herd health and communication. The frequency distribution of games across subject areas was similar between veterinary medical education and human medical education (Table 4).

**Table 5:** Table detailing the main data items in 13 publications of digital educational games in veterinary medicine identified in a scoping review of game-based learning in veterinary education.

Reference	Name	Country where the game is used	Study population	Game description	Game element^*^	Subject area	Access
Arauz et al., 2021 (28)	Neuropathology iBook (NP-iB)	Federation of Saint Kitts and Nevis	DVM students^2^ (2nd year)	A gamified learning tool created in iBook consists of 10 chapters, each representing a unique case. The objective of each chapter is to reach a diagnosis by answering questions based on the review of relevant medical history, clinical pathology, and autopsy findings.	R, G, C, F	Neuropathology	Could not be accessed
Irabor et al., 2023 (26)	Territory Game	Belgium	DVM students	Students are given an empty map and informational cards (via Google Slides) about a region (e.g., veterinary establishments, animal species) and collaborate to create a business plan for a veterinary practice.	R, G, I, C, F	Professional veterinary management	Could not be accessed
Klit et al., 2020 (41)	Professional Pig Practice	Denmark	Not tested on students but intended for veterinary and agriculture students	A digital game where the player takes on the role of a veterinarian at a pig farm, solving tasks that simulate real-life responsibilities during an on-farm visit.	R, G, C, F	Herd health	Could not be accessed
Nessler et al., 2021 (29)	Actionbound^1^	Germany	DVM students (2^nd^ to 4th year)	A digital scavenger hunt featuring neurological cases, where players perform diagnostic tests, answer questions, and work toward diagnosing and treating a virtual patient.	R, G, C, F	Clinical neurology	Partly, Actionbound is available, but the “neurology bounds” was not found
Nolan et al., 2019 (30)	Virtual Oncology Clinic	USA	DVM students (3rd year)	The player takes on the role of a veterinarian in an animal hospital, solving medical scenarios from anamnesis uptake to diagnosis and treatment.	R, C, F	Clinical oncology	Membership restrictions apply
Putra et al., 2022 (31)	Smart Sparrow^1^	USA	DVM students (3^rd^ and 4^th^ year)	The game consists of interactive modules: four focused on specific topics (e.g., skin lesion identification, laboratory tests) with questions to answer, and six patient simulation modules where players solve cases.	R, C	Clinical dermatology	Could not be accessed
Dorrestein et al., 2023 (27)	Veterinary DialogueTrainer (VDT)	Finland, Sweden	Bovine practicing veterinarians	The player participates in a digital role-play with a virtual dairy farmer, navigating different scenarios by selecting predefined interaction options and receiving scores based on the outcomes of the conversations.	R, G, C, F	Communication	Demo: https://en.dialoguetrainer.app/join/JVABAQAX. Full version requires an account.
Starke et al., 2021 (32)	LamenessTrainer	UK	DVM students (3^rd^ year)	An online platform where the player watches videos of a horse in motion and identifies if, and on which limb, the horse is lame. Points are awarded for correct answers, while incorrect answers result in “losing lives.”	R, G, C, F	Clinical science	Available at: http://www.lamenesstrainer.com/
López-Úbeda et al., 2022 (37)	Kahoot!^1^	Spain	DVM students (1st year)	Kahoot is an online platform for creating quizzes. The specific quiz consisted of five multiple-choice questions, each with four options and one correct answer. Students were scored based on accuracy and response speed, with a scoreboard displaying the top scores for each question and the overall total.	R, G, I, C, F	Physiology	Kahoot is available for all. The specific quiz could not be accessed.
Balogh, 2014 (33)	Virtual exam room	Not reported	Not tested but intended for veterinary students	A virtual clinic where the player examines patients, interprets diagnostic tests, and decides treatment. The case progresses based on the player’s actions, with feedback provided depending on the patient’s condition.	R, G, C, F	Clinical science	Could not be accessed
Mosley et al., 2019 (35)	SureWash	UK	DVM students (1^st^ to 4^th^ year)	A physical unit with an overhead camera and screen that uses camera recognition to assess handwashing. The player receives feedback and a score based on the completion of each handwashing step.	R, G, C, F	Hand hygiene	Requires purchase of physical product
Byron et al., 2014 (34)	Case Manager	USA	DVM students (4^th^ year)	An online platform featuring various cases where the player identifies a patient’s problem, considers differential diagnoses, and makes decisions on diagnostic tests and treatments within a budget. The case evolves based on the player’s actions, with feedback and scores provided based on decisions and responses to questions.	R, G, C, F	Clinical and problem-solving skills	Requires log-in at Ohio State University
Rawson and Quinlan, 2002 (36)	Acid/Base Primer	USA	DVM students (1^st^ year)	A program featuring cases on various acid–base balance disturbances. The player is given all relevant patient information, including blood gas. Analysis, and answers questions to interpret the condition (e.g., identifying the type of disturbance and its potential cause).	R, C, F	Physiology	Could not be accessed

^1^The software used to create the game is listed when the game itself does not have a specific name.

^2^Doctor of Veterinary Medicine (DVM) or equivalent.

^*^Presence of game elements as reported in the publication. R, rules; G, goals; I, interaction with others; C, challenge; F, feedback or outcomes.

The games varied greatly in nature; however, some sort of problem-solving was an important element for all. This was unsurprising, as problem-solving was one of the key game elements used to identify a game in this study. The problems to be solved ranged from creating a business plan for a veterinary clinic (26) to improving communication with farmers (27), with several games emphasizing decision-making or diagnosing disease in clinical settings based on anamnestic and/or diagnostic information (28–34). A short description of each game is included in Table 5.

Information on how to access the games was often incomplete or absent (Table 5). While some games provided partial access, such as test cases or demo versions, only one game, the *Lameness Trainer*, was fully accessible (32). As we did not perform comprehensive searches beyond the information provided in the articles, it is possible that other games may be available with additional effort.

### 3.4 Evaluation of games

Among the 13 articles reviewed, 10 included some form of evaluation of the educational games (26–32, 34–36). Of these, six studies evaluated cognitive learning outcomes by comparing student performance to either pre-test scores, repeated scoring or a control group (27, 28, 31, 32, 35, 36). Additionally, eight studies investigated affective outcomes, such as students’ perceptions of motivation, confidence, or performance (26–32, 34). Two studies measured the total time spent interacting with the application or game as an outcome variable (28, 32), and Nessler et al. (29) recorded the time to completion of different levels.

The *Interactive Neuropathology iBook* was evaluated using a post-test-only control group design, comparing student performance to a control group (28). They found no statistically significant differences in neuropathology-related questions between the groups and, somewhat unexpectedly, lower scores on other questions in the test group (28).

*The Territory Game* was assessed through individual and group responses to questions, as well as focus group interviews (26). The evaluation emphasized student perceptions regarding game features, confidence, and competency development. Results suggested that students perceived increased autonomy, and participants reported that engaging with the game enhanced their understanding of decision-making complexities. Similar to the *Territory game,* the study by Nessler et al. (29) also used questionnaires to ask for various aspects of the conduction of the game, *Actionbound*, such as time to download different game features and walking distance (as this game involved GPS assisted outdoor navigation in the forest). Students were asked to respond to questions after completing each “bound” (level). The evaluation also compared the experience of playing the game indoors versus outdoors, as well as the time taken to complete levels in each setting. The authors concluded that the game was perceived as both enjoyable and engaging, well-suited for case-based learning, and that outdoor play could further enhance student motivation.

In the study by Nolan et al. (30) the game *Virtual Oncology Clinic* was designed to allow students to engage in medical scenarios within a virtual hospital environment. The study placed significant emphasis on gathering student feedback through questionnaires, focusing on concrete suggestions for improving the game’s modules. However, the evaluation of students’ confidence in diagnosing and treating patients was limited to a comparison between those who played the initial version of the game (2014) and those who played the improved version (2015), without the inclusion of a separate control group. Another game focusing on clinical skills was the *Lameness Trainer*, which targeted the assessment of lameness in horses (32). This study evaluated using a multifaceted approach that included assessments of student perceptions, learning outcomes, and user behavior (32). Student perceptions were measured in terms of perceived difficulty, while learning outcomes were evaluated through various methods, including the comparison of students’ accuracy in classifying lameness before and after training. Additionally, user behavior was analyzed by measuring the time taken to complete a game module. The authors reported a positive effect on learning outcomes, particularly in the accurate identification of subtle forelimb lameness, as a result of engaging with the training (32).

In the study by Putra et al. (31), students (*n* = 28) were divided into two groups: one group was given access to the learning modules (*Adaptive Learning Modules in Veterinary Dermatology*), while the other group did not use them. The impact on learning outcomes was assessed by comparing pre- and post-test scores on a multiple-choice test that included questions requiring both basic recall and clinical reasoning. The authors report a significant improvement in favor of the group that used the learning modules (31). In addition to evaluating learning outcomes, the study also explored student perceptions of the modules. Feedback was collected on several aspects, including the quality of the design, ease of technology use, clarity of instructions, and the perceived increase in confidence in veterinary dermatology.

The *DialogueTrainer (VDT)*, described by Dorrestein et al. (27) is another simulation-based game; however, it differs significantly in scope. This game takes the form of a digital role-playing scenario with avatars, designed to enhance students’ communication skills. The study assessed the relationship between the number of attempts at the game scenarios and students’ communication skill scores, finding that, in general, communication skills improved with repeated attempts. Additionally, the researchers gathered feedback on participants’ willingness to recommend the VDT to others. Similarly, Mosley et al. (35) utilized both the number and duration of attempts as behavioral variables in their study of the *SureWash* system, a tool developed to train hand hygiene skills and implemented across several student cohorts. In this study, the number of attempts was used as a measure of exposure (i.e., interaction with the system) and analyzed for its association with hand hygiene performance, as assessed by the SureWash score. However, after accounting for user ID and class year, the authors found no statistically significant association between exposure and hand hygiene performance (35).

## 4 Discussion

This scoping review provides a characterization and comparison of publications exploring the use of game-based learning in veterinary and human medical education. Additionally, it offers a more detailed overview of the studies specifically focused on veterinary education.

More than half of the publications in this review described games within the clinical sciences. These games did not exclusively target hands-on clinical skills; many were designed to enhance cognitive abilities and decision-making processes in clinical scenarios (clinical reasoning). In veterinary medicine, the learning of clinical skills traditionally involves interaction with live animals. However, López-Úbeda and García-Vázquez (37) mention that stricter policies regarding the use of animals in teaching, and consequently a need to replace their use, might be a driver behind the implementation of pedagogical innovation. As emphasized by Molgaard et al. (38) veterinary graduates are licensed for practice without requiring additional practical training, thus they can practice across all aspects of veterinary medicine and animal species immediately following graduation. This contrasts with graduated medical doctors who gain practical experience under supervision through mandatory internships. The need for effective training on real world scenarios is therefore even more critical to veterinary students compared to medical students.

In the present study, we found that 10 out of 13 articles on veterinary education reported some form of evaluation of the game. Most evaluations relied on student perceptions such as perceived improvement in specific areas (e.g., clinical reasoning), increased motivation or willingness to recommend the game to others. Nevertheless, several studies also included measures of cognitive outcomes (learning outcomes). However, these were often assessed through repeated scoring, either across multiple attempts within the GBL or through pre- and post-course assessments. This is a limitation as the inclusion of a relevant comparison group allows changes in performance to be interpreted relative to an alternative educational condition rather than to pre/post change alone. Only two studies described the inclusion of control groups that did not interact with the game (28, 31). In Arauz et al. (28) adoption of the GBL activity was voluntary, and the groups were at the end of the course identified as users vs. non-users based on multiple choice questions embedded in the course test. Thus, no randomization or systematic allocation procedure were implemented. In the study by Putra et al. (31) students were allocated to a test group and a control group using a matched block design based on pre-test performance. More generally, the GBL activities described in the included articles were often evaluated in small samples and within a single class or cohort. In addition, participation in the evaluations was often voluntary introducing the risk of selection bias and in some cases further reducing power as the analysis was based on a subsample of students. Another limitation, relevant to the interpretation of this review, is that several articles were primarily concerned with describing the games themselves and outlining detailed features, rather than evaluating their effect on student motivation or learning.

A further issue identified in this review was the limited accessibility of described games. Only a few games were available for testing, and in most cases the access information was incomplete, restricted or absent. This limited accessibility hinders the sharing of learning resources and makes it difficult for educators to explore, evaluate or adopt these game-based learning resources in their own teaching. Furthermore, it reduce the potential for replication and building of evidence regarding their effect. More transparent reporting of access and maintenance of games would increase the practical value and potentially also the uptake of digital educational games in veterinary education.

Among the studies reporting cognitive outcomes, the majority described either positive results or no significant differences. Overall, the studies indicated that students generally had a positive perception of incorporating games into education. Affective outcomes, such as students’ self-reported perceptions of learning or increased confidence in handling specific scenarios, were frequently measured. Some evaluations also focused on feedback directly relevant to game development but less generalizable to other contexts. Examples include tracking the number of attempts and time spent interacting with the game (27, 29), students’ willingness to recommend the game (27) or specific elements like the avatar’s facial expressions or patient budgets in a virtual clinic (30). Furthermore, it is important to note that the inclusion of game elements does not inherently guarantee engagement or motivation for all learners. For instance, Mosley et al. (35) reported low levels of engagement in their study, with only 13% of students choosing to interact with the system.

Previous research has also yielded mixed results regarding the impact of game-based learning on students’ learning outcomes. For example, in a recent review and meta-analysis of gamification in educational settings Huang et al. (16) found a small to medium-sized positive effect on student learning outcomes. Similarly, Sailer and Homner (39) reported a comparable effect on cognitive learning outcomes in their meta-analysis, though they noted that the effects were less stable for motivational and behavioral outcomes. Genty et al. (15) concluded in their review that, overall, the use of serious games appears to be at least as effective as traditional methods, and in many studies, even more effective for improving knowledge, skills, and satisfaction.

Establishing a clear definition of a digital game was essential for determining which publications to include in this review. While reaching a universally accepted definition of “a game” may be nearly impossible, we contend that most individuals engaging in an activity are likely to have their own perception of whether or not they consider it a game. In an editorial, David Crookall (10) discuss the challenges of defining terms *simulation*, *game* and *serious game*. His observation from over a decade ago remains relevant: “The debate will surely continue, and we will be blessed—and confused—with as many definitions as authors who care to offer their thoughts on the matter” (10). A more recent and comprehensive review of the definitions of gamification and related terms is provided by van Gaalen et al. (18). They highlight how different authors often use varying terms for the same concept or apply the same term to different concepts, underscoring the challenges posed by the lack of uniformity in definitions (18).

In the present review, we restricted inclusion to digital games, as these represent a relatively new area of educational practice and our aim was to map what has been described specifically in veterinary medical education. However, this restriction narrowed the evidence base and excluded relevant analog educational games, such as board games, card games, and role-play activities, which are also used in veterinary and medical education. Simulations present an additional layer of complexity, as their classification as games is frequently debated. Some simulations incorporate elements typical of gameplay, such as point scoring, competition, or problem solving, while others lack these features. Despite this, many individuals may still perceive such simulations as games. Van Gaalen et al. (18) suggest that simulations are best understood as learning activities that inherently include some game-like intention but do not explicitly utilize game elements. Consistent with the aims of a scoping review we used a broad inclusion approach to better capture the range of relevant studies in a field where terminology is inconsistent and overlapping. However, this decision may have led to the inclusion of borderline cases and we also acknowledge that our classification relied solely on the features reported by the authors, as we did not ourselves play the included games. Thus, the findings should be interpreted as describing a broad landscape of digital game-based and game-like learning activities. We also included simulations when they met the inclusion criteria by incorporating at least two game elements (e.g., competition or point scoring). This consistent approach ensured that all articles were evaluated using the same criteria, and allowed us to include a broader range of educational activities that align with the principles of digital GBL.

The games included in this scoping review met our operational definition of a game if describing at least two out of five predefined game elements. Commonly described game elements were some sort of *challenge*, typically involving some sort of problem solving, e.g., to solve a medical case or reach the correct diagnosis. Another frequent element was *feedback or outcome*, usually in the form of rewards, achievements, or scores provided by the game to communicate the results of players’ actions and indicate progress. At the same time, we found that the games described in the reviewed articles were highly diverse in terms of design elements and learning objectives. For example, the game *Actionbound* utilizes GPS-based technology, enabling students to engage in outdoor activities while solving a case (29). In contrast, *The Territory Game* employed a more conventional setup, using Google Slides and PDF slides to facilitate learning (26), while Mosley et al. (35) used a physical unit, namely a handwashing simulator, offering an entirely different type of learning activity. These examples illustrate the wide variation in learning activities with totally different game elements and learning goals. This diversity makes it challenging to draw generalized conclusions about the overall experience and effects of using games in veterinary education. Both the reviews by Huang et al. (16) and Sailer and Homner (39) emphasize the significant heterogeneity among studies, which underscores the complexity of research on educational games. Acknowledging this heterogeneity, both these meta-analyses investigated possible moderators of effect. Huang et al. (16) investigated the role of contextual elements (e.g., subject area or educational level) and specific game elements (e.g., quests/missions, leaderboards, collaboration) as moderators of gamification effects. They found that the effectiveness of using games for educational purposes is influenced by both these contextual factors and the game elements employed highlighting the importance of considering these variables when designing and implementing gamified learning interventions. Sailer and Homer (39) found that inclusion of game fiction and social interaction were significant moderators of the effect of gamification on behavioral learning outcomes.

Addressing heterogeneity by examining moderators becomes challenging when the number of included studies is small, either due to the niche nature of the research area or a lack of available studies as there may not be enough data. In the presented review we therefore describe a narrative synthesis of the included articles. A similar approach was used in the context of medical education by Gentry et al. (15) due to the considerable heterogeneity across studies. They noted that the variability in populations, interventions, comparisons, and outcomes made it challenging to conduct a meaningful meta-analysis. Similarly, Rutledge et al. (13) argue that rather than focusing solely on whether gamification in medical education leads to improved learning outcomes, future research should emphasize understanding *how* and *under what conditions* gamification is most effective. They recommend a thoughtful approach to integrating game design elements into educational frameworks (13). In the context of the present review, the relatively narrow field of veterinary medicine education and the limited number of included studies precluded deeper analysis of the effects of contextual or game elements. This highlights the need for more educators in veterinary and medical sciences to publish the use of educational games and include detailed accounts of their approaches, clearly describing the contextual factors, game elements, and associated outcomes. Such contributions would enable more robust evidence synthesis and provide clearer recommendations for effectively implementing game-based learning approaches within the veterinary medical education.

The most significant limitation of this study is the likely underreporting of educational games and the exclusion of grey literature, which might contain additional relevant reports. Many educational games are likely never documented in the scientific literature, and although information about such games may exist online, no comprehensive repository of digital games for learning in veterinary science currently exists to the authors’ knowledge. This lack of centralized resources poses a challenge for sharing educational tools across institutions and countries, limiting collaboration and broader adoption in the field.

Another challenge was establishing clear criteria to define and recognize *game-based learning activities using a digital device in an educational context*. Subsequently, determining whether the activities described in the articles met our definition of a digital game was sometimes difficult. Additionally, the wide range of terminology used for these activities posed further challenges. While we used comprehensive search strategy and accessed three scientific databases, we missed some articles as three publications included in the review were identified from the reference lists of other articles, despite the activities meeting the criteria for a game in this review. The reason was that authors had not used the terms “game” or “gami*” in their papers. This suggests that additional relevant articles may exist but are difficult to identify due to inconsistent terminology, as there is no universally accepted term to encompass such activities in search strategies. Furthermore, although we relied on previous scientific literature, the concept of “game elements” remains subject to debate. Even with relatively clear criteria, the decision-making process was sometimes challenging and may have resulted in the exclusion of relevant publications. To address this, we adopted a liberal approach to inclusion, incorporating any publication where there was uncertainty about eligibility. However, despite these efforts, the inherent challenges of reviewing a niche and underreported research area remain a limiting factor.

This scoping review highlights the limited reporting and descriptions of games in veterinary education. The included studies predominantly focused on clinical sciences and were most often applied at the undergraduate level. However, the inconsistent approaches to game evaluation, the wide variation in game elements, and differing applications and learning outcomes make it challenging to draw definitive conclusions about their overall effectiveness. Despite these limitations, existing research indicates that digital educational games are generally well-received and may enhance learning. Further studies are needed to explore the specific conditions, design features, and mechanisms that maximize their impact in veterinary education.

## 5 Conclusion

This scoping review revealed significantly more reports of educational games in human medical education compared to veterinary education. In both disciplines, the majority of reports focused on undergraduate-level use. Specifically, we identified 13 publications describing the use of games in veterinary education, spanning various subject areas, with clinical sciences being the most prominent. Most games in veterinary education included some form of evaluation, typically through repeated scoring of student performance or by assessing affective outcomes, such as students’ perceptions of learning and other aspects of the gaming experience. Information on how to access the games was frequently missing.

The limited number and wide diversity of studies in veterinary education make it challenging to generalize the effects of game-based learning. Nevertheless, existing evidence suggests that digital educational games are generally well-received and may enhance learning outcomes. Further research is needed to identify the specific conditions, design features, and mechanisms that maximize the effectiveness of digital educational games in veterinary education.

## Data Availability

The original contributions presented in the study are publicly available. This data can be found here: https://doi.org/10.17605/OSF.IO/Z4WNJ.
